# Optimal design of stimulus experiments for robust discrimination of biochemical reaction networks

**DOI:** 10.1093/bioinformatics/bts585

**Published:** 2012-10-09

**Authors:** R. J. Flassig, K. Sundmacher

**Affiliations:** ^1^Otto-von-Guericke University, Process Systems Engineering, Universitätsplatz 2, ^2^Max Planck Institute for Dynamics of Complex Technical Systems, Process Systems Engineering Sandtorstr. 1, D-39106 Magdeburg, Germany

## Abstract

**Motivation**: Biochemical reaction networks in the form of coupled ordinary differential equations (ODEs) provide a powerful modeling tool for understanding the dynamics of biochemical processes. During the early phase of modeling, scientists have to deal with a large pool of competing nonlinear models. At this point, discrimination experiments can be designed and conducted to obtain optimal data for selecting the most plausible model. Since biological ODE models have widely distributed parameters due to, e.g. biologic variability or experimental variations, model responses become distributed. Therefore, a robust optimal experimental design (OED) for model discrimination can be used to discriminate models based on their response probability distribution functions (PDFs).

**Results**: In this work, we present an optimal control-based methodology for designing optimal stimulus experiments aimed at robust model discrimination. For estimating the time-varying model response PDF, which results from the nonlinear propagation of the parameter PDF under the ODE dynamics, we suggest using the sigma-point approach. Using the model overlap (expected likelihood) as a robust discrimination criterion to measure dissimilarities between expected model response PDFs, we benchmark the proposed nonlinear design approach against linearization with respect to prediction accuracy and design quality for two nonlinear biological reaction networks. As shown, the sigma-point outperforms the linearization approach in the case of widely distributed parameter sets and/or existing multiple steady states. Since the sigma-point approach scales linearly with the number of model parameter, it can be applied to large systems for robust experimental planning.

**Availability**: An implementation of the method in MATLAB/AMPL is available at http://www.uni-magdeburg.de/ivt/svt/person/rf/roed.html.

**Contact**: flassig@mpi-magdeburg.mpg.de

**Supplementary information**: Supplementary data are are available at *Bioinformatics* online.

## 1 INTRODUCTION

Mathematical models of complex biological processes provide the basis for systems understanding. They are of vital importance for generating predictions of systems behavior based on hypothesized mechanisms. In the case of limited experimental access to biological systems, models can help to find missing links and provide a tool to aggregate existing knowledge and data. In this work, we focus on mathematical models in the form of coupled ordinary differential equations (ODEs), which are being used to describe dynamics of biochemical reaction networks, e.g. signal transduction, metabolic or genetic regulation, on a deterministic, (semi-)mechanistic basis. Here, scientists are often facing limited or even contradicting knowledge about the underlying mechanisms, confined experimental possibilities, large biological variability as well as measurement noise. This leads to largely distributed model parameter sets, which in combination with several plausible alternative model structures renders model-based prediction highly uncertain. Therefore, model-based experimental design (e.g. optimal stimulus or additional experimental readout selection) is used to generate experiments that yield optimal experimental data to (i) reduce the spread in the model parameters (=optimal parameter identification) and/or (ii) reduce the pool of plausible models (=optimal model discrimination).

Much work on optimal experimental stimulus design (OESD) for biological systems focuses on information maximization with respect to parameter identification (Balsa-Canto *et al.*, 2008; Bandara *et al.*, 2009; Heine *et al.*, 2008; Raue *et al.*, 2010; Schenkendorf *et al.*, 2009a). Here, for a given pool of plausible ODE models, OESD is used to find experimental conditions that reduce the parameter uncertainties and thus model response variabilities. The methods used to quantify parameter uncertainties and model response variabilities include linearization, sigma-points (Julier *et al.*, 2000), profile likelihood (Raue *et al.*, 2009) and Markov chain Monte Carlo (MCMC, Geyer, 1992; Vanlier *et al.*, 2012). An OESD aiming at model discrimination for biological systems has been addressed by few authors (Apgar *et al.*, 2008; Melykuti *et al.*, 2010; Skanda and Lebiedz, 2010). Although measurement noise has been included, a rigorous consideration of model response variabilities due to distributed parameters has been missing so far. For chemical reaction kinetics and biotechnology, there exists some work on designing dynamic stimuli for the purpose of model discrimination (Asprey and Macchietto, 2002; Box and Hill, 1967; Kremling *et al.*, 2004). As has been illustrated, the consideration of model response variabilities strongly improves the designed experiments and experimental data quality (Chen and Asprey, 2003; Donckels, 2012; Michalik *et al.*, 2010). Therefore, linearization of the system’s parameter mapping has been used. However, by using the so-called Sigma-Point method (Julier *et al.*, 2000), (Heine *et al.*, 2008), (Schenkendorf *et al.*, 2009a) showed that the performance of a linear OED for best parameter estimation is rather poor for nonlinear systems having widely distributed parameters.

An experimental design aimed at model discrimination is typically generated at a point where existing data do not provide further discriminative information for a pool of competing, validated and identifiable models. With these models, robust experimental designs can be generated, which presumably yield data with optimal discrimination information. Since predictions with ODE models depend on the model parameters, identifiability is important as it ensures the existence of a unique solution to the parameter estimation problem and, consequently, unique model predictions (Raue *et al.*, 2009). A non-identifiable model would yield non-unique model predictions under altered experimental design conditions, as there exists a set of several solutions to the parameter estimation problem. Robustness of the experimental design is achieved by considering (i) pure uncertainty about the model itself, (ii) distributed model predictions that arise from distributed model parameters and (iii) design variabilities (e.g. variations of the applied stimulus) during the conduction of the experiment.

In this work, part (ii) of design robustification for nonlinear models is considered, focusing on computational efficient and accurate estimation of the nonlinear propagated parameter probability distribution function (PDF) in an optimal control framework. We suggest using the sigma-point method as an alternative to the classical linearization approach. Therefore, both approaches are presented, applied and compared in the light of OESD for robust model discrimination, assuming perfect experimental conduction. In the following section, we describe the essential parts of our design approach, including (i) ODE modeling of biological reaction networks with and extended interpretation of distributed determinism, (ii) the model overlap as a PDF-based discrimination criterion, (iii) linearization and sigma-point methods and (iv) two numerical approaches to solve optimal control problems. Then, using two numerical examples, we formulate the optimization problem and benchmark the linearization approach against the sigma-point approach (Section 3).

## 2 METHODS

### 2.1 Dynamic modeling of biochemical reaction systems

ODEs provide the modeling basis to describe the dynamics of biochemical reaction networks. The dynamics of the internal states 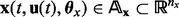, e.g. protein concentrations, is determined by the solution of an initial value problem of the form
(1)
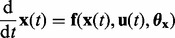

with initial system states 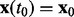 and right-hand side function 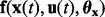 describing biologic interaction mechanisms, which depends on the system states **x**(*t*), (multiple) inputs **u**(*t*) (=stimulus) and kinetic parameter set 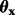. Assuming **f** to be Lipschitz in **x**(*t*), **u**(*t*) and continuous in *t*, the readout variables are determined by
(2)
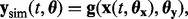

where the function **g**—assumed to be sufficiently smooth—relates the internal system states to the readouts of the experiment with corresponding readout parameters 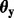, which together with dynamic parameters and initial conditions are merged into the model parameter vector 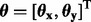, with redefined dynamic parameter vector 
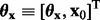
. The dynamic model defined by Equations (1) and (2) can be understood as a time-dependent mapping from the model parameter space 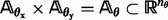 to the model output space 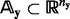,
(3)
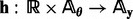

(4)
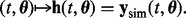



### 2.2 Distributed determinism

Although biological systems might follow deterministic rules, repeated measurements, even though with very accurate measurement techniques, will yield different results. The reasons for that are manyfold. Additionally to unavoidable measurement errors, biologic variability, i.e. systems with intrinsically distributed parameters, can induce a large spread in the transient dynamics and stationary behavior. In the case of the existence of multiple steady states, this spreading effect might even be more pronounced. Varying parameters during the measuring procedure and local parameter perturbations by non-stationary noise also contribute to a distributed measurement signal (Lorenz *et al.*, 2007). Complex, nonlinear models of biological systems might also behave chaotic, further contributing to distributed response measurements. Thus, the conventional sharp, deterministic system representation needs to be extended by the notion of distributed determinism, i.e. although the system might completely be deterministic, its perceived signals are distributed realizations of the underlying deterministic mechanisms. This can be done by considering the parameters, and hence, the model responses as random variables 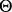 and **Y**, respectively, each characterized by a PDF. We point out that, within this interpretation, the system and hence the model is assumed to naturally possess distributed parameters. Consequently, a distributed response is not solely explained by additive measurement noise but also by other sources of variations, which may be represented as distributed parameter sets.

Let the model parameters be distributed according to some well-defined PDF 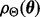, with 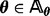 being a realization of 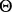. The PDF of the random model response **Y** at time *t* can be derived from the normalized integral over all possible parameter and corresponding response realizations, weighted with the parameter PDF, i.e.
(5)
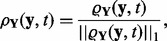

with
(6)
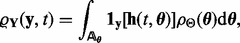

where 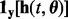 represents the indicator function
(7)
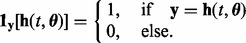



The normalization employs the 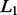-norm with respect to 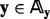. Note that **y** represents an arbitrary possible realization of **Y** in 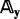, whereas 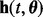 describes the model response at time *t* for a fixed stimulus time course 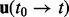 given parameter realization 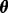. Consequently, for every single point in time, the shape of Equation (5) is determined by the parameter PDF, the choice of model [Equations (1) and (2)] and stimulus time course (=experimental design). Consequently, the discrimination process can be robustified by discriminating competing models based on their model response PDFs [Equation (5)], accounting for variabilities in the parameters and model-specific mapping to the response space.

### 2.3 Robust design criteria for model discrimination

Here, we present the model overlap as a robust discrimination criterion, measuring dissimilarities of model response PDFs used to rate the discriminative power of a design during optimization. We define the *general model overlap* as the probability product kernel used in vector machine learning to measure statistical distances for the sake of discriminative learning (Jebara *et al.*, 2004). Following Jebara *et al.*, (2004), the probability product kernel of two multivariate PDFs 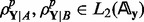 is defined as the scalar product
(8)
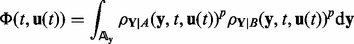

with the densities being raised to some power 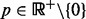. It provides a bounded, positive-definite measure (Jebara *et al.*, 2004) of similarity between distributions on the set 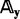, whereas the parameter *p* controls the weighting of regions with small versus large densities.

From this, the average overlap of the time course is
(9)
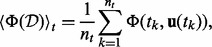

where 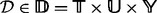 represents the experimental design within the feasible design region 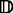, encompassing for instance selection of discrete measurement time points 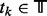, stimulus design 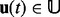 and readout design 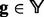. Using the model response PDFs 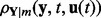 from Equation (5) for two competing models, 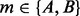 in Equation (9) provides us with a measure of average model dissimilarities. For *p* = 1, the overlap is the expected model response probability of model *A* under model *B* and vice versa [Equation (8)]. In this case, assuming one of the models to be true, the overlap yields the expected likelihood of the other model depending on the experimental design 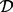. Consequently, an optimal model discrimination design 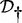 minimizes Equation (9). Singh (1998) proposed to use Equation (8) with *p* = 1/2—known as Bhattacharyya’s affinity between distributions—for discriminating nonlinear regression models. It is closely related to Hellinger’s distance, which represents a symmetrized approximation to the Kullback–Leibler divergence (Topsoe, 2000). In this work, we use the overlap as defined in Equation (9) with *p* = 1, which we refer to as *model overlap*, as this directly represent the time-averaged, expected likelihood of one model under the other. In this form, it has been applied by Lorenz *et al.*, (2007) to discriminate dynamic models based on the distributional fit performance. In Schenkendorf *et al.*, (2009b), it is used to optimize an initial condition for a substrate uptake model to discriminate two competing kinetic approaches.

### 2.4 Estimation of nonlinear PDF mapping

If the solution 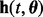 can be obtained in closed form, it is straightforward to derive the model response PDF for a given parameter PDF using Equation (5). However, in most of the cases, the model response 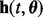 for a specific parameter realization is obtained by numerical integration. Here, besides random sampling techniques based on Monte-Carlo simulations, the approximate model response PDF may also be obtained by deterministic sampling, e.g. by simple enumeration, optimized Latin hypercubes of the parameter space and application of Equation (5). For an infinite number of samples, the true model response PDF can be constructed from these samples, which can be used for a subsequent evaluation of Equation (9) to judge the quality of a given design. Such procedures become computational inefficient for increasing number of model parameters and cannot be used in an optimization framework. Therefore, an approximation has to be made. From initial data, one may obtain accurate estimates for the true parameter PDF, e.g. by MCMC sampling, which can be approximated by unimodal normal PDFs 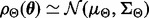, possibly multivariate. The model response PDF can also be approximated by 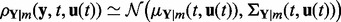. Then, the integration in Equation (8) can be performed to yield the approximate overlap
(10)
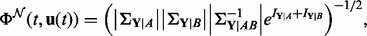

where

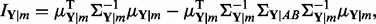


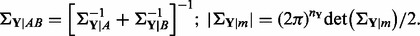

Here, *m* = {*A, B*} represents the model index, 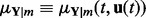 the 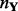-dimensional true mean and 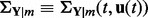 the true variance-covariance matrix of the corresponding model response PDFs, which both depend on the model structure *m*, measurement time point *t*, stimulus **u**(*t*) and readout selection. For details about the derivation, see for instance, Jebara *et al.* (2004). For 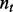 discrete measurement time points 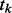, the approximate mean overlap is then
(11)
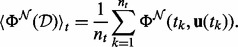



**Fig. 1.**
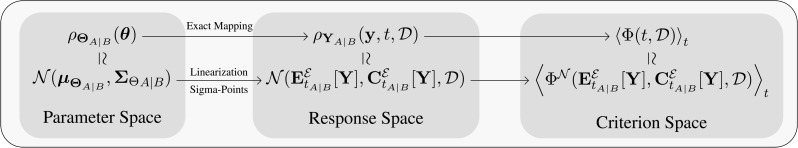
Approximation of nonlinear PDF mapping

This approximation dramatically reduces the computational costs as only the two first statistical moments (i.e. expectation and variance-covariance) need to be estimated. The task of solving Equation (5) to obtain model-response PDFs and subsequent integration of Equation (11) to evaluate the discriminative power of a given design in an optimization framework is then reduced to estimate the time course of mean vector 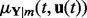 and variance–covariance matrix 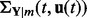 of two model response PDFs for given parameter expectation 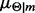 and variance–covariance 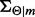 (Fig. 1). As true mean and variance–covariance of the parameters are unknown, these are replaced by their estimates, i.e. 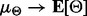 and 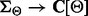, which we will do in the following. Note that for skewed PDFs one should apply a transformation, e.g. Box–Cox or inverse hyperbolic sine transformation to achieve normality of the PDF (Box, 1964; Johnson, 1949). In this way, our presented robust OESD method is not restricted to normal PDFs only.

Estimates of response expectation and variance–covariance can be obtained by linearizing the system at additional computational costs that scale linear with the number of parameters using forward-sensitivity analysis. But this approach can become suboptimal or yield even misleading designs (Section 3.2). On the additional expense of 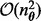 estimates may be improved by a quadratic approximation of the system, which may become infeasible for larger systems, as do Monte Carlo-based approaches. Worst-case approaches yielding a minimax design have also been proposed to take model response variabilities into account (Walter and Pronzato, 1997; Skanda and Lebiedz, 2012). However, these are in general NP-hard (Du and Pardalos, 1995). The sigma-point method has an additional computational expense of 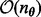, which compares to linearization.

#### 2.4.1 Estimation based on linearization

The classical approach to estimate model response variabilities is linearization of the nonlinear model mapping 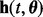 with respect to the parameters. The linearization of the model response is given by applying the chain rule to Equation (2)
(12)
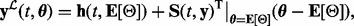

with response sensitivity matrix 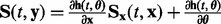 and state-sensitivity matrix 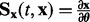, which can be obtained by solving
(13)
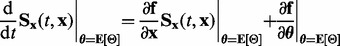

with initial condition 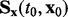 along the systems dynamics, which is known as the forward-sensitivity matrix equation. The additional computational effort is of order 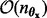, as only 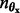 additional ODEs have to be solved in Equation (13), since 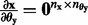. One may also formulate an adjoint system to derive the state sensitivities in a backward manner or use numerical differentiation.

Having determined the parameter sensitivities of the system, the linear estimates of expectation and variance–covariances of the model response PDF can be calculated to yield
(14)
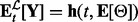

(15)
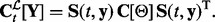



For nonlinear models, the estimate of the expectation is typically biased, i.e. 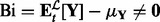, and errors are introduced at second and higher orders. The quality of the predicted variance–covariance cannot readily be judged as the errors are of fourth and higher order, whereas the contributions depend on the system. Notice that the linear design approach yields a local estimate in the parameter space, i.e. parameter-dependent coexisting stable states will be missed, resulting in significant estimation errors in both moments (Section 3.2). The estimators are exact for linear systems, as higher-order terms vanish.

#### 2.4.2 Estimation based on sigma-points

Julier *et al.* (2000) introduced the sigma-point method for advanced Kalman filtering and state estimation. It is based on the idea that with a fixed set of parameters (sigma-points), it is easier to approximate a nonlinearly transformed PDF by a Gaussian distribution than the nonlinear transformation itself. Julier *et al.* (2000) show that expectation and variance–covariance of a random variable **Y**, given by a transformation 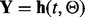, possibly nonlinear, of a random variable 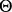 with expectation 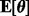 and variance–covariance 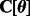 can be estimated according to the following procedure:

1. Select 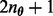 sigma-points in the original domain according to

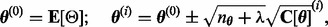

where 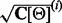 is the *i*th column of the square root of the variance–covariance matrix.
2. Propagate these points through the model

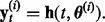

3. Estimated expectation and variance–covariance of the transformed variable based on the sigma-points are given by the linearly weighted sums
(16)
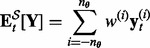

(17)
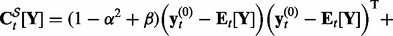


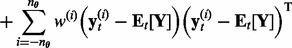

with weights 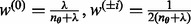 and 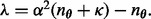

According to Julier *et al.* (2000), the errors of the expectation estimate is of fourth and higher order, whereas the variance–covariance estimates have an error of fourth and higher order. This, however, only holds for scalars, i.e. 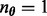, as pointed out by Gustafsson and Hendeby (2008). For 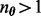, the sigma-point parameters (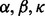) can be used to tune the estimated moments by including a priori knowledge about the PDFs, i.e. 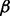 and 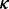 allow to account for higher-order moments of the parameter PDF and should be set to 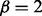 for an initial Gaussian, whereas for 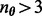 one should choose 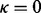. Further, 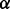 controls the sigma-point spread and should lie within 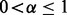 (Julier *et al.*, 2000). The sigma-point has several advantages:

- no need to calculate derivative information (neither Jacobian nor Hessian have to be available or need to exist), which makes this method numerically robust and applicable to a wide range of system classes,
- use of curvature information of the system,
- deterministic sampling method with computational effort that scales linearly with the number of distributed variables, i.e. 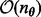,
- since each sigma-point is independently propagated, parallelization can easily be applied to speed up estimate calculation of the transformed expectation and variance–covariance.

### 2.5 Robust optimal stimulus design

The problem of finding an optimal stimulus design can be stated as an optimal control problem. Given a nonlinear dynamic system of the form Equations (1) and(2) and corresponding parameter set (expectation and variance–covariance), an optimal stimulus is an admissible control defined over an interval 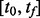, say experimental time window, at which a cost function assumes its infimum (or supremum) with the set of all admissible controls. Robustness of such a control with respect to distributed model responses can be achieved by incorporating expectation and variance–covariance into a robust design criterion (e.g. model overlap). Within the sigma-point approach, variabilities in the stimulus conductions can also be accounted by interpreting a design **u**(*t*) as a time-dependent mean of a distributed variable **U**. Then, for a design **u**(*t*), the spread in the model response PDF is determined by the propagation of sigma-points given by mean and variance–covariances of (i) model parameters and (ii) stimulus. The problem of finding an optimal control may be solved by (i) Hamilton-Jacobi-Bellman, (ii) variational or (iii) NLP-based approaches (Nevistic nonlinear programming, 1997). We use the following two direct NLP-based approaches (Biegler, 2007), which can easily be combined with the methods discussed in Sections 2.4 and 2.4.1 for mapping distributed parameters onto the design criterion:

- *Direct sequential approach:* A control vector parameterization in combination with numerical integration of the model equations. This approach is suited for design problems without nonlinear path constraints and stable behavior with respect to variations in the control and parameters.
- *Direct simultaneous:* A full discretization of the problem, e.g. control vector and state/response vector parameterization, is based on orthogonal collocation on finite elements. If the design problem includes nonlinear path constraints, this solution approach can be beneficial, because feasibility of the solution is ensured at the collocation points of each finite element.

Both NLP approaches are typically non-convex, i.e. there exist several local and possibly one global optimal design solution. Therefore, resulting solutions to the NLP problem are local optima. Global optimality of the design can be achieved—but is not ensured—by (i) performing local optimizations from different initial starting points and/or (ii) deterministic/stochastic/heuristic global optimizers (Floudas and Gounaris, 2009; Horst *et al.*, 2000; Zabinsky, 2003). We point out that optimal design solutions need not necessarily be global in real-life applications. Local optimal solutions can be very close to the global solution with respect to the design criterion. Therefore, non-convexity allows to account for further experimental constraints—restricting the degrees of freedom in the design space—without losing, e.g. discriminative power. In the applications, we use global stochastic and multistart optimizations to avoid a biased comparison between linearization and sigma-point approach.

## 3 APPLICATION

### 3.1 Application I: signaling cascade

The highly conserved mitogen-activated protein kinase signaling cascade (Pearson *et al.*, 2001) with two different hypothesized negative feedbacks is used as a nonlinear test system for benchmarking the two design approaches with respect to estimation accuracy and design quality. Multistep signaling cascades are enrolled in many signal-transduction processes of cells to sense and react to external signals. Upon an external stimulus, e.g. growth factor, hormones or stress signals, cascades transduce the signal from the cell membrane to the nucleus to start different cell programs. The respective ODE systems—adapted from Behar *et al.* (2007)—of two model candidates 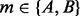 that describe the change in protein concentration of the phosphorylated forms are

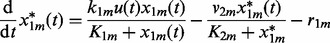


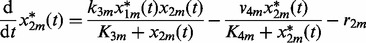


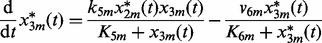

with model *A*:

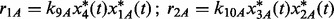


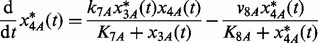

and model *B*:

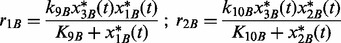


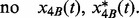


For both models, we assume

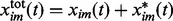


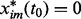

with the total concentration of each species 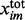 as an additional model parameter and 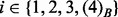.

The measurement response signals are defined as
(18)
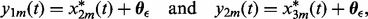

where 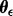 represents additive measurement noise, which we assume to be normally distributed with zero mean and variance 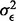. We assume that the response signals can be measured at 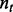 specific time points. Based on an initial stimulus design, we adjust the model parameters so that both model responses match up to a small error, which does not allow to prefer one over the other model, to mimic the starting point of an OESD for model discrimination. Identifiability of the models has been checked with the software tool DAISY in combination with global sensitivity analysis (see Supplementary Material, Bellu *et al.*, 2007; Sobol’, 1993). Because biological systems often follow a log-normal distribution, we apply a log-normal transformation to the response to improve the normal approximation used in the estimation approaches for the response PDF (Sections 2.4 and 2.4.1). Therefore, we redefine the response signal Equation (18) used for the overlap calculation as
(19)
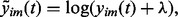

with *i* = 1, 2, 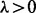. For each model, we assume that all dynamic parameters are log-normally distributed, with nominal value being the expectation 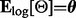 and diagonal covariance matrix 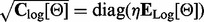, with scaling parameter 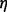. The measurement noise is typically independent on the stimulus design and thus held constant at 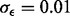. The sigma-points for the log-normal parameter PDF are obtained in the following way: In the parameter space, we derive the normal equivalents of log-normal expectation and covariance to calculate the normal sigma-points, which we then exponentiate (see Supplementary Material). The log-normal sigma-points are propagated through the model, including Equation (19), to obtain the normal estimates via Equations (16) and (17). In the following, we drop the tilde from the redefined response signal in Equation (19).

Following the direct sequential approach, the stimulus (single input) is parameterized as

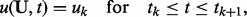

with 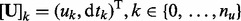, whereas 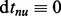. Here, 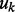 represents the amount of stimulus between the time point 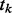 and 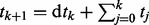. If for the last time point, we have 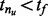, we put 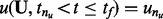. On the other hand, if 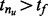, the design is given a penalty. Depending on the estimation method 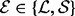, the resulting optimization problem for discriminating between models *A* and *B* is formulated as an NLP problem (see Supplementary Material for details), i.e.
(20)
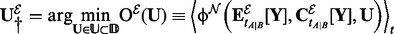

subject to systems dynamic, additional constraints and method to estimate 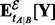 and 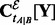.

The number of optimization parameters is 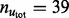, which allows 20 stimulations 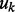 with 19 stimulus durations 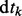. Because the problem is non-convex, we use a hybrid optimization strategy, consisting of the evolutionary-based CMA-ES algorithm (Covariance Matrix Adaptation Evolution Strategy Hansen and Ostermeier, 2001), in combination with a subsequent gradient-based optimizer. Because of the stochastic nature, the hybrid optimization is performed 40 times for each parameter variance level, which we derive from the scaling parameter 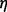. The benchmark is based on a Monte Carlo verification of the resulting optimal stimulus designs. For each optimal design, the overlap, including expectation and variance–covariance of the model responses, is calculated based on sampling the parameter space 10^4^ times for each model and corresponding optimal stimulus design (see Supplementary Material for details). The relative mean-squared error (MSE) of the moment estimates are given by

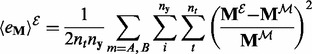

with 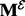 being the moment estimates of the best designs (expectation 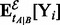, variance–covariance split into variance 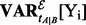 and covariance terms 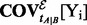). The Monte Carlo reference is represented by 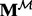.

In Table 1, we see that for all parameter variance levels, both methods have negligible relative MSE in the mean response estimates (maximal MSE: 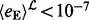; 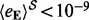). In contrast, the relative MSEs for linearization increases with parameter variance levels up to 0.03 for the variance and 0.18 for the covariance estimates. Here, the sigma-point approach performs better with maximal relative MSE of the variance 0.007 and covariance 0.096. In this application, both approaches estimate mean responses of the models very well, although the maximal MSE of the sigma-points is still two orders of magnitudes smaller than the maximal MSE for linearization. For the (co)variance estimates, the sigma-point approach consistently outperforms linearization approach for increasing parameter variance level.

**Table 1.** Relative MSEs of moment estimates and overlap (scaled 10^5^) of the best designs based on linearization/sigma-point estimation and corresponding Monte Carlo verification

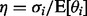		0.01		0.1		0.2		0.3		0.4	
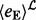	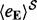	0	0	0	0	0	0	0	0	0	0
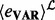	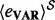	0	0	0.002	0	0.001	0	0.01	0.007	0.03	0.007
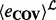	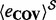	0	0	0.02	0.002	0.059	0.015	0.106	0.046	0.181	0.096
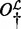	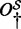	0.2	0.2	2	2	2	2	2	3	3	3
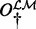	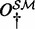	0.2	0.2	2	2	2	2	3	3	4	3

In the lower part of Table 1, we compare the discriminative power of the resulting designs for different parameter variance levels. Comparing the Monte Carlo verifications, we see that, for small variances, both methods yield designs that have the same discriminative power (
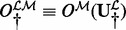
versus 
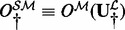
). However, for widely distributed parameters (starting at 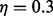) sigma-point based designs perform up to 1.3 times better than linearization-based designs and their estimates coincide with the MC validation, which is not the case for linearization. For both methods, optimization time for one design is 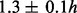 on a standard desktop computer (4 GB RAM, 3 GHz quad core processor) and mainly determined by the optimizer itself, whereas the validation time (10^4^ MC samples) of a single optimal design is 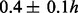.

**Fig. 2.**
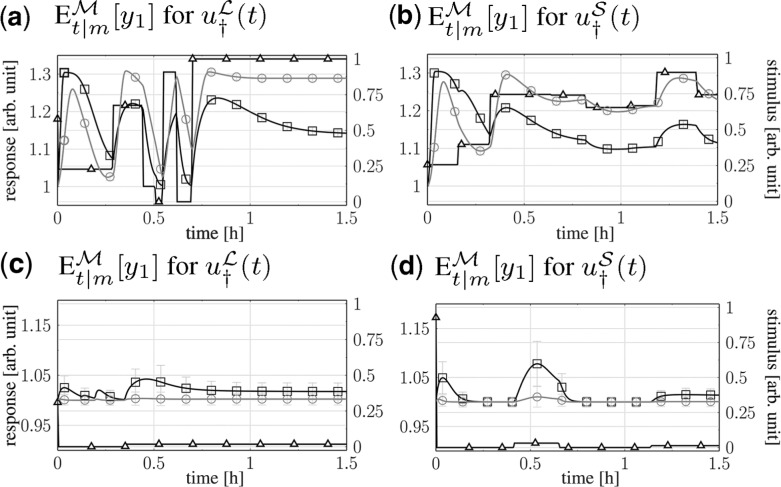
Expected responses of models *A* (crossed square), *B* (crossed circle) on log-scale for optimized stimuli 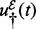 (crossed triangle) and corresponding 
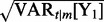
 bands [first component only, **(a)/(b)** 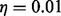, **(c)/(d)** 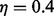]

In Figure 2, we show the best designs based on the estimates for two levels of parameter variances and the first response component. For small parameter variances, both designs yield the same discriminative power. For widely distributed parameters, we see that both methods tend to minimize the amount of stimulation, as this results into little response variances at maximal distances between expected model responses. Although the linear design is characterized by a strong stimulation right at the beginning, with a subsequent plateau of little stimulation, the sigma-point design starts with an even stronger initial pulse, followed by two small pulses.

### 3.2 Application II: Schlögl model

In this section, we compare the performance of the nonlinear design based on the sigma-point approach to the linear design in the presence of multiple steady states. The Schlögl model is a canonical example of a biochemical reaction system exhibiting bistability (Schlögl, 1972). It describes an autocatalytic, trimolecular reaction that, for instance, occurs in post-translational modifications of signaling proteins. Two model alternatives for the rate of concentration change of species *x* are given by

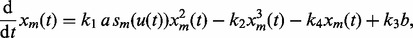


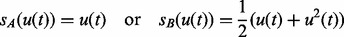

where we assume the four kinetic parameters 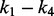 as well as the system parameters a and b to be distributed 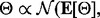
diag(η**E**[Θ])^2^), the initial condition as 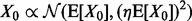. The mean parameter values are taken from Vellela and Qian (2009), which also discuss the consequences of bi-(multi)stability for biological modeling in the light of non-equilibrium thermo- and stochastic dynamics. The model alternatives simply differ in the input layer 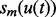. Parameters *a* and *b* represent the concentration of two reaction partners *a* and *b* of species *x*, which both are in constant exchange with a material reservoir. For an initial, suboptimal experiment with stimulus 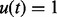, models *A* and *B* cannot be distinguished, given 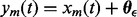 to be the response signal, where 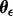 represents additive measurement noise with zero mean and 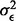. The stimulus is thought to control the concentration in the reservoir of species *a* to find an optimal discriminative stimulus, whereas subsequent stimulations can only be applied after a minimal time period has passed to account for possible control limitations (see Supplementary Material for details). Such nonlinear constraint optimization can efficiently be solved within the direct simultaneous approach, which we apply using orthogonal collocation on 100 finite elements (each with three collocation points) to discretize control and system states. The objective of the resulting non-convex NLP problem is the same as in Equation (20), however, subjected to different constraints, i.e. system dynamics in form of a nonlinear algebraic equation system and additional constraints (details see Supplementary Material). For the linear design strategy, we implement the sensitivity Equation (13) and corresponding constraints. For the sigma-point design, the constraints have to simultaneously hold for all 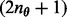 sigma–points. The solver AMPL in combination with the optimizer CONOPT is used to solve the aforementioned NLP problem (Drud, 1994). For a given optimization setup (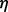 and estimation method), the solution takes about 2 min on a standard desktop computer. Because CONOPT yields local solutions, the optimization is performed for 1000 different randomized initial designs for a given optimization setup, from which the best solution is selected.

**Fig. 3.**
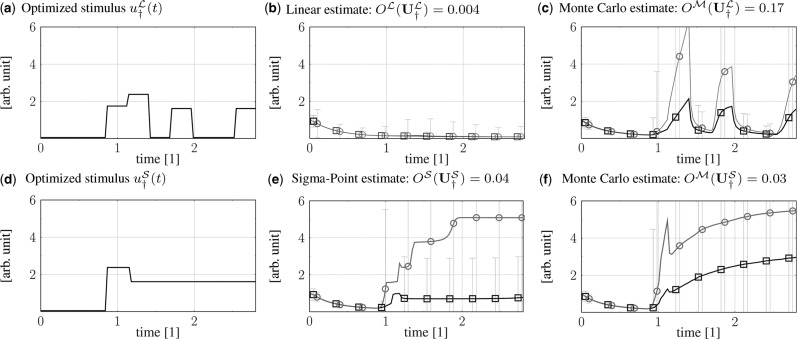
Upper part: **(a)** Optimal stimulus 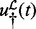 (bold line) and corresponding 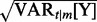 bands based on linearization and corresponding linear **(b)** and MC **(c)** estimates of expected responses of models *A* (crossed square) and *B* (crossed circle) for 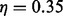. Lower part **(d)-(f)**: sigma-point based results

In Figure 3, we show the resulting stimuli designs for 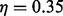 based on linearization and sigma-point estimation. Reexamination of the optimized linear design with MC simulations reveals a large underestimation of the estimated overlap: 
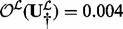
 versus 
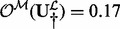
, i.e. misleading discriminative power by two orders of magnitude. The local estimation property of the linear approach yields a highly biased expectation and underestimation of the variance with relative MSE of 0.44 for the expected response and 6.66 for the variance [see Fig. 3, estimated response of model *B*, (b) versus (c)]. The sigma-point based design (d) in Figure 3 has a relative MSE of 0.15 for the expected response and 0.38 for the variance. The overlap estimate of the sigma-point design closely matches the MC validation [
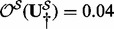
 versus 
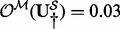
]. Further, the sigma-point design performs 5.7 better than the linear design 
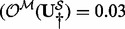
 versus 
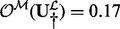
). As can be seen in Figure 3 (e,f), the non-local propagation property of the sigma-points enables the optimizer to find a stimulus that mostly drives model *B* to the upper steady state.

## 4 CONCLUSION

Biological variability in combination with experimental measurement noise results into widely distributed response signals, which is one of the main challenges when modeling biological system deterministically with nonlinear ODEs. The parameter set needs to be extended to a parameter distribution. In this way, natural variability in the dynamic parameters as well as measurement noise can be readily accounted for. However, an exact quantification is computationally expensive and infeasible in an optimization framework for large systems. Therefore, approximate descriptions of the PDFs and nonlinear mapping process between parameter and model response space have to be used. Here, we have presented a nonlinear design approach based on the sigma-points within the application of model-based OESD aimed at model discrimination. We illustrate the application and performance in combination with two numerical approaches from optimal control for two nonlinear model examples. Using the overlap as a robust design criterion based on the model response PDFs, we show that in the case of nonlinear models with widely distributed parameter PDFs, the sigma-point predictions and designs consistently outperform a linear design approach. In the case of bi-(multi)stability, we further illustrate the benefit of the nonlocal propagation property. Finally, the sigma-points come with several numerical advantages, including linear scaling of the numerical costs with respect to distributed parameters and derivative free estimation of nonlinearly mapped expectation and variance–covariance. The latter property allows applying a robust OESD to dynamic models that have non-smooth right-hand side functions, e.g. cybernetic models of cellular metabolism (Ramkrishna, 1982).
